# QitanTech Nanopore Long-Read Sequencing Enables Rapid Resolution of Complete Genomes of Multi-Drug Resistant Pathogens

**DOI:** 10.3389/fmicb.2022.778659

**Published:** 2022-03-23

**Authors:** Kai Peng, Yi Yin, Yan Li, Shangshang Qin, Yuan Liu, Xiaorong Yang, Zhiqiang Wang, Ruichao Li

**Affiliations:** ^1^Jiangsu Co-innovation Center for Prevention and Control of Important Animal Infectious Diseases and Zoonoses, College of Veterinary Medicine, Yangzhou University, Yangzhou, China; ^2^Institute of Comparative Medicine, Yangzhou University, Yangzhou, China; ^3^Key Laboratory of Advanced Drug Preparation Technologies, School of Pharmaceutical Sciences, Zhengzhou University, Zhengzhou, China; ^4^Center for Disease Control and Prevention of Sichuan Province, Chengdu, China

**Keywords:** antimicrobial resistance, microbial genomics, long-read sequencing, multi-drug resistant (MDR) bacteria, QitanTech sequencing

## Abstract

Advancement of novel sequencing technologies facilitates modern life science and medicine unprecedentedly. Exploring complete genome sequences of bacteria by long-read sequencing technology is significant for microbial genomics research. However, third-generation long-read sequencing technologies are available with limited choices, which generate technological barrier to scientific research. Recently, a novel QitanTech nanopore long-read sequencing technology has emerged in China, but the potential application and performance were unexplored. Herein, we comprehensively evaluated the feasibility of the emerging sequencing technology in assembling complete genomes of MDR pathogens. The results showed that 500 Mbp QitanTech nanopore sequencing data could be generated within 8 h in one flow cell with the standard library preparation method. The mean read length, longest read length, and mean read-level accuracy of QitanTech sequencing data were 6,041 bp, 57,037 bp, and 81.50% (LAST)/81.40% (Minimap2), respectively. Two routine assembly strategies including long-read assembly and hybrid assembly enable the achievement of complete bacterial genomes. The accuracy of assembled draft bacterial genomes with QitanTech long-read data could be improved up to 99.9% dramatically by polishing using accurate short-read data. Furthermore, the assembled bacterial genomes cover accurate structures of complex resistance plasmids harboring critical resistance genes such as *tet*(X), *tmexCD-toprJ*, and *bla*_VIM–2_, even the complex fusion MDR plasmid generated from homologous recombination. In conclusion, QitanTech nanopore sequencing, as a nanopore long-read sequencing technology launched in China, could be a good option for investigation of complex bacterial genomes. More potential applications based on this novel platform warrant investigations.

## Introduction

Deciphering the genetic code of organisms with DNA sequencing technologies is an important technical innovation for understanding the basis of life in all forms. The initial method for DNA sequencing was developed by Sanger and termed as Sanger sequencing forty years ago, also called first-generation sequencing technology (Sanger and Coulson, 1975). Sanger sequencing was used routinely until now due to its high accuracy and easy accessibility. However, the low throughput of first-generation sequencing limited its ability to sequence large genomes and thus promoted the development of next-generation sequencing (NGS) technology, which is also called second-generation sequencing technology. In the past two decades, the application of second-generation sequencing technology has made unprecedented achievements and accounted for most of the sequencing data nowadays. NGS costs less and generates high-throughput data with high sequencing accuracy (Heather and Chain, 2016). Various second-generation sequencing technologies including Illumina (Solexa) sequencing (Voelkerding et al., 2009), Roche 454 sequencing (Margulies et al., 2005), Proton/PGM sequencing (Yuan et al., 2013), and SOLiD sequencing (Mardis, 2008) competed with each other for many years but finally dominated by Illumina due to its low cost and high accuracy. Subsequently, the drawbacks of short-read second-generation sequencing limited their specific applications such as genome assembly, which pushed the innovation of sequencing technologies, and third-generation sequencing came out 10 years ago. The current third-generation sequencing is dominated by PacBio single-molecule real-time (SMRT) sequencing technology (Eid et al., 2009) and Oxford Nanopore Technologies (ONT) nanopore sequencing technology (Clarke et al., 2009; Eisenstein, 2012; Loman and Quinlan, 2014; van Dijk et al., 2018). The characteristic of third-generation sequencing was able to generate long-read data without PCR amplification of fragmented DNA molecules, but on the basis of original DNA molecules. Long-read sequencing technology has irreplaceable and significant advantages in genome assembly, large structural variation detecting, complex population analysis, and so on (van Dijk et al., 2018). In addition, third-generation sequencing developed by ONT generated longer reads but higher error rates compared with sequencing data produced by PacBio SMRT, even so, the portability and easy availability of ONT nanopore sequencing platforms are revolutionizing the genomics research of life science comprehensively. However, apart from ONT nanopore sequencing technology, no other alternative nanopore sequencing technologies are available worldwide.

Obtaining complete bacterial genomes is a very important step to further perform research in terms of basic microbial biology, microbial pathogenesis, antimicrobial resistance, and microbial genomics. However, it was difficult to construct accurate complete genomes with short-read sequencing data due to the abundance of repeat sequences, especially for the multi-drug resistant (MDR) bacteria (Klassen and Currie, 2012). Generally, the complex repeat sequences in bacterial genome were mainly insertion sequences distributed in MDR regions, plasmids, and resistance islands, which were important mediators of horizontal gene transfer. Meanwhile, these insertion sequences enhanced the frequency of gene exchange between bacterial genomes, resulting in many MDR islands and pathogenicity islands (Thomson et al., 2004; Partridge et al., 2018). It is especially essential to analyze the structure and position of these regions for understanding the evolution of bacterial genome. At present, deciphering complex bacterial genomes by combining third-generation long-read sequencing with second-generation short-read sequencing data has become commonplace (Kamada et al., 2014; Ashton et al., 2015; Liu et al., 2016; Li et al., 2018). However, owing to the limited choice and high cost of third-generation sequencing, which prevented them from becoming as popular as second-generation sequencing. Recently, an emerging nanopore sequencing technology developed by QitanTech in China is developing rapidly and has introduced an early access program for users to evaluate its application in various research settings. We participated in the Early User Program of QitanTech and evaluated its performance in resolving genomes of MDR bacteria comprehensively. In this pilot study, we aim to evaluate the ability of QitanTech long-read sequences in assembling complete bacterial genomes, and evidence that QitanTech sequencing technology is a potential contender in the long-read sequencing arena.

## Materials and Methods

### Samples Preparation, DNA Extraction, and DNA Quality Control

To evaluate the ability of QitanTech sequencing in obtaining the complete genome of MDR bacteria, six MDR bacteria with different species, spanning a wide range of genome sizes and GC contents, were selected as the test strains (Table 1). The complete genomes of six isolates have been obtained via hybrid assembly strategy on the basis of ONT nanopore sequencing and Illumina sequencing data. First, these strains were cultured in Luria-Bertani (LB) broth. Then, the genomic DNA was extracted using the FastPure Bacteria DNA Isolation Mini Kit (Vazyme) following the manufacturer’s instruction. Briefly, 1 ml of bacterial culture in logarithmic growth period was collected by centrifugation at 12,000 rpm/min. The bacterial pellet was lysed with proteinase K and treated with RNAase. Then, the genomic DNA was extracted using the column extraction method. The purity of genomic DNA was evaluated by NanoDrop 2000 and gel electrophoresis. The concentration of genomic DNA was determined accurately using a dsDNA Broad-Range Assay kit on the Qubit^®^ Fluorometer.

**TABLE 1:** The alignment characteristics of QitanTech nanopore sequencing raw data against the six complete genomes.

Strain	Species	Minimap2			LAST		
		General correct rate (%)	Mean mapping quality	Mean coverage (X)	General correct rate (%)	Mean mapping quality	Mean coverage (X)
KP18-2073	*Klebsiella pneumoniae*	80.71	54.62	161.14	80.81	91.79	162.68
KP18-2110-2	*Klebsiella pneumoniae*	80.19	54.29	115.16	80.31	91.26	116.29
RGT40-1	*Klebsiella pneumoniae*	80.81	54.31	93.11	80.92	91.64	94.03
XM9F202-2	*Acinetobacter variabilis*	83.79	55.39	207.24	83.88	92.91	208.56
ZF2	*Proteus cibarius*	82.11	53.63	120.01	82.13	90.00	121.20
ZXPA-20	*Pseudomonas putida*	80.81	54.27	154.70	80.92	91.35	156.05
Mean	/	81.40	54.42	/	81.50	91.49	/

### Genomic DNA Sequencing and Data Acquisition

Genomic DNA of the six strains was sent out for short-read DNA sequencing on Illumina Hiseq X platform using the PE150 strategy at the GENEWIZ (Suzhou, China). Adapters and low-quality bases of short-read sequences were trimmed using Trimmomatic v0.36 (Bolger et al., 2014). Clean fastq reads with about 150 X genome size were generated for each sample. Meanwhile, the extracted genomic DNA samples of the six strains were sent out for nanopore single-molecule long-read sequencing at QitanTech (Beijing, China). The library preparation and sequencing process on the basis of QitanTech technology platform were performed as follows. First, the total DNA was sheared into sizes ranging from 6 to 20 kb using g-TUBE (Covaris). Then, 150–300 fmol of fragment DNA were preformed end-repair and dA-tailing. In detail, 47 μl of fragment DNA, 7 μl of End-prep reaction buffer, 3 μl of End-prep Mix, and 3 μl of DNA Repair Mix were mixed and incubated in a thermocycler at 20°C for 10 min at first followed by another incubation at 65°C for 10 min. The end-prepped DNA was purified using beads and added sequencing adapters with T4 Ligase. Next, adapted DNA was purified again. Finally, adapted and purified DNA was quantified using Qubit as abovementioned and then sequenced using the QitanTech first nanopore sequencer QNome-9604. Both short-read and long-read data were directly obtained with fastq format for further analysis.

### QitanTech Long-Read Quality Evaluation

We firstly evaluated the quality of total sequences of six samples generated from QitanTech nanopore sequencer according to the read length N50, the average base quality score, mean read length, and so on using NanoPack v1.25.0 (De Coster et al., 2018), which is a toolkit dedicated to evaluate the quality of long-read sequences. The data qualities of different samples were evaluated using Nanoplot of NanoPack, and multiple samples comparison of data quality analysis was performed using NanoComp of NanoPack. The alignment characteristics of QitanTech original data were generated by minimap2 (Li, 2018) and LAST (Frith et al., 2010) and then summarized and visualized by Qualimap2 v2.2.1 (Okonechnikov et al., 2016).

### Genome Assembly and Polishing

At present, the complete bacterial genomes were usually obtained using short-read data to correct the draft genomes assembled by long-read data or using hybrid assembly strategy combining both short-read and long-read data. Here, we used both methods to assess the ability of QitanTech long-read data in assembling complete bacterial genomes. The short-read sequences were assembled using SPAdes v3.13.1 with the coverage cutoff value of 30 (Bankevich et al., 2012). There are no specifically assembly tools for the long-read data generated by QitanTech sequencer due to it has just been developed and is in the initial testing phase. Hence, all long-read sequences assembly tools we used in this study were developed for ONT nanopore sequences or PacBio SMRT sequences. We selected Flye v2.8-b1674 (Kolmogorov et al., 2019) and Canu v1.6 (Koren et al., 2017), two most commonly used long-read data assemblers, to assemble QitanTech long-read sequences. Then, we used Pilon v1.22 (Walker et al., 2014) and NextPolish v1.0.5 (Hu et al., 2020) to carry out genomes polishing using high-accuracy second-generation sequencing data. After that, we used Unicycler v0.4.8 (Wick et al., 2017) to perform hybrid assembly based on QitanTech and Illumina data. We filtered QitanTech long-read data using Seqkit v0.8.0 (Shen et al., 2016) and then perform hybrid assembly to eliminate the adverse impact of some short reads in the long-read data on hybrid assembly results.

### Genome Integrity and Accuracy Assessment

Firstly, we counted the reports generated by different assemblers and used Bandage v0.8.1 to visualize the assembly results (Wick et al., 2015). Then, we took the standard completed genome as a reference and used QUAST v4.6.3 to evaluate the integrity and accuracy of bacterial genomes obtained by different strategies (Gurevich et al., 2013). Subsequently, the accuracy of the genomes successfully assembled using QitanTech long-read sequencing data was evaluated by dnadiffer in the MuMmer toolkit v3.23 (Cornelius et al., 2021). Simultaneously, one strain with the highest sequencing depth was selected, randomly sampling sequences from QitanTech raw data using Seqkit (Shen et al., 2016) and reassembled genome to compare the influence of different sequencing depths on the final assembly results.

### Genome Annotation and Resistance Genes Identification

For bacterial genomes assembled using QitanTech sequences, the functional annotation was achieved using the RAST^1^ automatically and then modified manually (Aziz et al., 2008; Overbeek et al., 2014; Brettin et al., 2015). Antimicrobial resistance genes and insertion sequences were identified by ResFinder 4.1 and PlasmidFinder 2.1 in CGE services.^2^ The *cfr* gene in genome ZF2 was detected by PCR amplification with primers described in a previous study (Kehrenberg and Schwarz, 2006). BRIG v0.95 (Alikhan et al., 2011) and Easyfig v2.1 (Sullivan et al., 2011) tools were used to visualize the fusion plasmid comparison.

## Results and Discussion

### The Basic Information of the Six Bacterial Strains

The genomic characteristics, such as genome sizes and GC contents, have a strong influence on assembling complete bacterial genomes. In addition, some complex regions consisting of many resistance genes, insertion sequences, and rRNA operon in bacterial genomes are difficult to resolve by short-read sequencing (Thomson et al., 2004). To comprehensively assess the ability of the novel sequencing technology in complete bacterial genomes acquisition, we selected four reported MDR bacteria and two clinical MDR *Klebsiella pneumoniae* to perform QitanTech nanopore sequencing. All complete genomes of these strains have been obtained with hybrid assembly strategy combining ONT nanopore sequences and Illumina sequences as previous description (Li et al., 2018). The four strains were a *tmexCD1-toprJ1*–positive *K. pneumoniae* RGT40-1, an *Acinetobacter variabilis* XM9F202-2 co-harboring *tet*(X3) and *tet*(X15) (Li et al., 2021b), a *Proteus cibarius* ZF2 carrying a chromosomal *tet*(X6)-bearing integrative and conjugative element (ICE) (Peng et al., 2020), and a *Pseudomonas putida* ZXPA-20 carrying a *tmexCD1-toprJ1* and *bla*_VIM–2_–positive megaplasmid (Li et al., 2021a; Supplementary Table 1). These MDR strains evolved from general bacteria by acquiring foreign resistance genes and posed a great threat to public health (Partridge et al., 2018). Deciphering the genetic features of these bacteria is important for understanding the formation and transmission of MDR regions in bacterial genomes. Many complex MDR genetic structures, including plasmids, ICEs, and metaplasmids, have been identified in the four strains. Hence, it is critical to evaluate whether the novel QitanTech nanopore sequencing technology could be utilized to obtain the complete genomes of such strains. Apart from the four strains, another two MDR *K. pneumoniae* strains were recovered from clinical settings. Understanding the genetic features of such high-risk *K. pneumoniae* is important to guide clinical treatment and trace the source of infection.

### QitanTech Long Reads Data Acquisition

QitanTech announced its first product prototype nanopore sequencer QNome-9604 in September 2020 and invited potential academics to participate in its Early User Program. The Early User Program claimed that QitanTech sequencing technology was based on the principle of nanopore strand sequencing with its proprietary sequencing chemistry and 500-Mb data with median single-read accuracy over 80% could be generated within 8-h sequencing time. We sent six bacterial genomic DNA with minimal requirement of 1,000 ng to Qitan. Libraries were prepared by technical team of QitanTech using sequencing reagent kit Qeagen-8 without any PCR procedure, and 40-fmol library with volume of 200 μl was loaded on to each sequencing flow cell Qcell-3841. Then, the flow cell was assembled onto the QNome-9604 sequencer which was connected to a GPU-equipped computer via USB cable. Experiment control parameters were operated by software QNOME v1.3. Raw data were generated within minutes after the flow cell temperature reached the desired sequencing condition and then were base called with QNOME based on deep neural network algorithm. Sequencing data in fastq format (Q7 filtered), together with a sequencing report, were sent back to us for downstream analysis.

### The Sequence Features of QitanTech Sequencing

**FIGURE 1:**
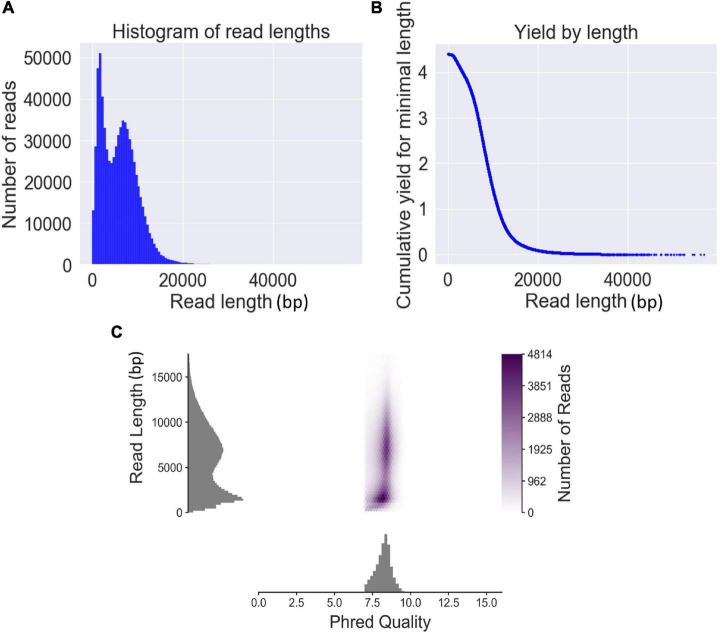
The read features of QitanTech nanopore sequencing. **(A)** The distribution of read lengths. **(B)** The relationship between read length and total bases. The unit of the ordinate is Gbp. **(C)** The plot shows relationship between read length and read Phred quality.

The genomic DNA of the six strains was sequenced using six separate sequencing chips (flow cells). A total of 727,854 reads with 4,397 Mbp were obtained from six sequencing chips, and the sequencing data volumes were ranging from 496 to 1,063 Mbp generated from each sample (Supplementary Table 2). The average read length and average read quality in *Q*-value of total sequencing data were 6,041 bp and 8.2, respectively. The read length N50 of the total QitanTech sequencing data was 8,314 bp. Read length distribution analysis found that the read length of sequencing data was mainly distributed in approximately 2 and 8 kb (Figure 1A). Aggregation of these short-read data about 2 kb was most likely caused by some high-copy small plasmids in bacterial genomes. Hence, we pointed out that the true read length of the sequencing data produced by QitanTech nanopore sequencer was about 8 kb on average, which was much longer than the read length of first- and second-generation sequencing data. Surprisingly, the throughput, mean read length, and *Q*-value of QitanTech raw reads were better than ONT nanopore raw reads generated by R7 flow cells initially released in 2014 (Ashton et al., 2015; Quick et al., 2015). At present, ONT sequencing has far better performance than QitanTech sequencing (Todd et al., 2018; Naushad et al., 2020), which even proposes a Q20 plan for sequencing accuracy. Even then, it is difficult for ONT nanopore sequencing to catch up with second-generation sequencing comprehensively in terms of sequencing accuracy within a short period of time. Therefore, both ONT nanopore sequencing and QitanTech nanopore sequencing technologies remain to make improvements especially in sequencing accuracy. As for PacBio SMRT, it generated reads with an average length about 15–20 kb and average accuracy greater than 99% (van Dijk et al., 2018). However, high cost in instrument setting and expensive maintenance of the sequencing equipment make it unaffordable for most researchers and research institutes, except large-scale genomics centers or bioinformatics companies (van Dijk et al., 2018). Subsequently, we observed that the number of reads decreases as the length of the sequence becomes longer (Figure 1B). In addition, according to the distribution of read length and read Phred quality, we found that there was no obvious relationship between the read quality and read length (Figure 1C). Although the best quality of read has a highest quality score up to 16, it is still meaningless as it is only one bp in length. The mean quality score of the top five longest reads (more than 50 kb) was greater than 8.2. This demonstrated that QitanTech nanopore sequencing would not generate more base mistakes with sequencing read length increasing. We found that the reads quality of ONT nanopore sequencing would be worse with the sequencing voltage increasing during multiple experiments based on one flow cell. The sequencing voltage is directly related to the speed of bases passing through nanopores. High voltage accelerated DNA molecules passing through nanopores, which resulted in a hazy change in the magnitude of the current in the nanopore captured by a sensor. Hence, we speculated that the base mistakes of reads generated by QitanTech nanopore sequencer might be influenced by the rate of bases passing through nanopores. Overall, the emerging sequencing technology performed well in read length and read quality. In addition, it is necessary to further evaluate the application of QitanTech nanopore sequencing data in bacterial genome assembly.

**FIGURE 2:**
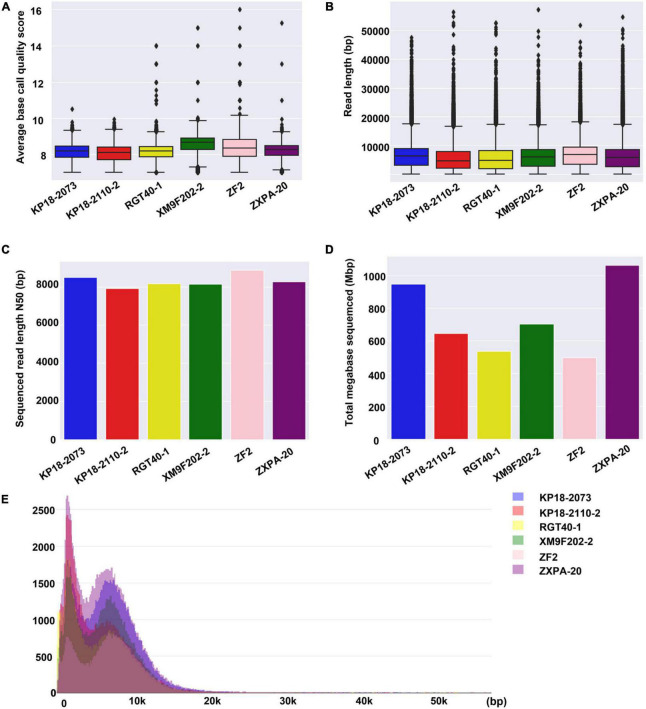
Base quality, read length, read length N50, total base, and distribution of read length for QitanTech sequencing of six strains. **(A)** Overview to show the average base Phred score of the six bacterial QitanTech sequencing data. **(B)** The read length distribution of QitanTech sequencing data of the six strains. **(C)** Comparing QitanTech read length N50 of the six strains. **(D)** Comparing throughput in Mbps of the six strains. **(E)** Histogram of read lengths of the six bacterial QitanTech sequencing raw data.

Subsequently, we compared the sequence features for six samples. It was found that there was no significant difference in the sequencing data of the six samples in terms of average base quality, read length and read length N50 (Figures 2A–C). In addition, the read length distribution of the six samples was similar (Figure 2E). However, the total bases of each sample generated from different sequencing chips were different with the same library preparation method and running time (Figure 2D). We obtained 1,063 Mbp data from sample ZXPA-20, which is more than twice the total bases of sample ZF2 (496 Mbp). The difference in total sequence data between each sample might be influenced by many factors, such as the DNA library quality and sequencing chips quality. Similar situations are also frequently found in ONT nanopore sequencing platforms (Ashton et al., 2015; Quick et al., 2015; Lu et al., 2016). At present, the sequencing data generated by one QitanTech nanopore sequencing chip were enough to assemble a bacterial genome (sequencing depth of more than 100×). However, QitanTech nanopore sequencing was in its infancy; thus, the throughput was much lower compared with current second- and third-generation sequencers. The throughput of QitanTech will increase as the improvement of sequencing technology, and it will certainly introduce sequencing indexes for multiple samples in a single run.

### The Alignment Homogeneity and Accuracy

**FIGURE 3:**
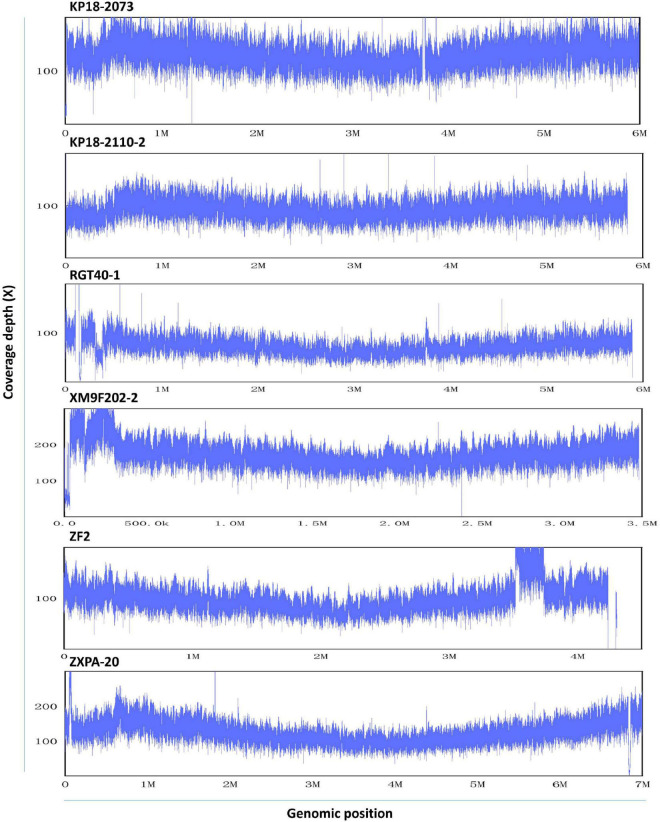
The sequence homogeneity of QitanTech nanopore sequencing. The ordinate indicates the sequencing coverage (X). The abscissa indicates the genome location.

To assess the homogeneity of QitanTech nanopore sequencing data, we mapped the QitanTech raw reads against the six reference genomes using LAST. We found that the uniformity of sequence coverage of the six genomes was fine (Figure 3). Apart from some plasmids, the chromosome sequencing coverage was relatively homogeneous. Plasmids were extrachromosomal DNA elements with the ability of self-replication, which usually had different copies (Carroll and Wong, 2018). Hence, it was unsurprising to us to observe a coverage discrepancy in plasmids with chromosomes. According to the results, QitanTech sequencing performed well and did not have a sequencing bias.

The accuracy of QitanTech nanopore sequencing derived from Phred quality score was a measure of the likely accuracy of the base called from base calling software, which did not represent the real identity of QitanTech raw reads relative to the standard complete genomes. Here, we calculated the read-level accuracy using alignments strategy. The six bacterial QitanTech nanopore reads were mapped to their reference genome using LAST and Minimap2. According to previous study, LAST performed better than other programs as it can align sequences with many mismatches and gaps (Ashton et al., 2015). Minimap2 was developed recently and was adept at dealing with ultra-long reads (Li, 2018). Comparing the mapping-derived accuracy of different alignments strategy showed slightly different estimates (Table 1). The mean accuracy computed from Minimap2 (81.40%) was lower than LAST (81.50%). In addition, the read-level accuracy of QitanTech nanopore sequences was better than initial ONT nanopore sequencing and PacBio sequencing with the same alignment method (van Dijk et al., 2018). The high base error rate of long-read sequencing data made them less useful for single nucleotide polymorphism (SNP) typing. However, the completeness of bacterial genomes could be enhanced using these long reads.

### The Genome Assemblies

Initially, we used SPAdes to *de novo* assemble the short-read data of the six genomes. Each of them was assembled into one genome scaffold with size similar to their reference genome. Subsequently, we analyzed the genome scaffold of each strain in detail. The longest contig in each genome scaffold were in length ranging from 150 to 564 kb. The average length of contigs of these scaffolds was in length ranging from 17 to 36 kb (Supplementary Table 3). Overall, short-read data can generate draft genomes of bacteria. However, it is difficult to recognize plasmid sequences and chromosome sequences using short-read data assembly strategy.

**FIGURE 4:**
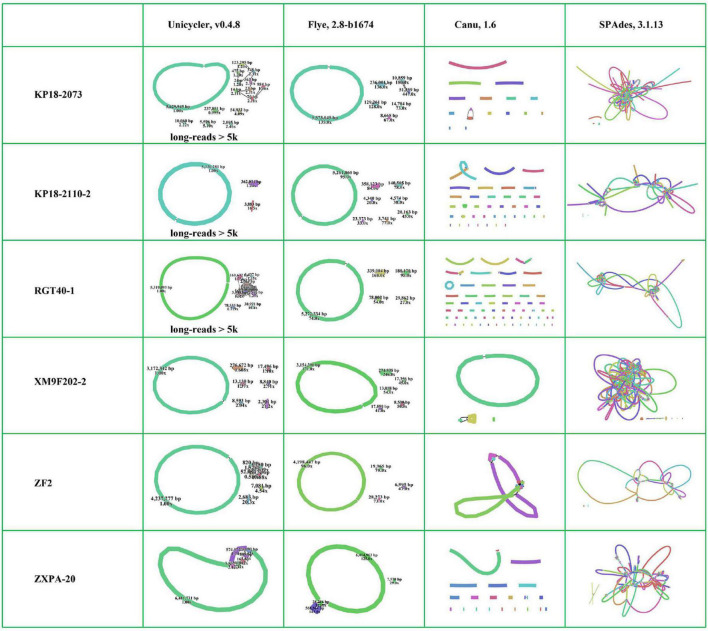
The assembly results of the six genomes using different methods. Every independent line indicates one assembly contig. The individual circular ring indicates circular chromosomes or plasmids.

Many tools have been developed for the genome *de novo* assembly and polishing for long-read data (Jain et al., 2016). Here, we selected Flye (Kolmogorov et al., 2019) and Canu (Berlin et al., 2015; Koren et al., 2017) assembling tools, which were the commonly used error-prone long-read assemblers, to perform long-read *de novo* assembly. The six bacterial genomes were almost assembled into complete maps using Flye. In contrast, most of them were assembled into many contigs with Canu (Figure 4 and Supplementary Figure 1). Canu performs error correction by finding the overlaps of the raw uncorrected reads first and then finds the overlaps between corrected reads to construct a draft genome (Koren et al., 2017). Flye firstly generates arbitrary paths from an unknown repeat graph, which called disjointigs, and then constructs an accurate repeat graph from error-riddled disjointigs (Kolmogorov et al., 2019). According to previous study, Flye could generate more contiguous and accurate assemblies from complex databases based on its algorithm compared with Canu (Kolmogorov et al., 2019). The six bacterial genomes were complex due to containing many insert sequences and exogenous genes. Obviously, Flye performed better than Canu in assembling such bacterial genomes with QitanTech nanopore long-read sequencing data. Then, we analyzed the genomes assembled with Flye in details. We find that the chromosomes of five strains were assembled into circular closed contigs except for strain ZXPA-20. In addition, most plasmids in the six strains were successfully assembled into circular contigs (Figure 4). Although the size of plasmids were much smaller than bacterial chromosomes, their structures were usually complex due to presence of many resistance genes, insert sequences, and repeat regions (Smalla et al., 2015). In most cases, accessing to complete MDR plasmids is often more difficult than the acquisition of complete bacterial chromosomes and sequencing reads length play a vital role in doing this. In general, the sequencing data generated from QitanTech nanopore sequencer performed well in the acquisition of complete bacterial genomes.

Owing to the low accuracy of long-read sequencing data, the accurate complete bacterial genomes were usually obtained by hybrid assembly combining long-read data and high accurate short-read data. Unicycler, a hybrid assembly pipeline for bacterial genomes, has been widely used since it was developed and it achieved accurate complete bacterial genomes recognized as gold standard (Wick et al., 2017; Li et al., 2018). To assess the function of QitanTech nanopore sequencing data with hybrid assembly strategy, we used Unicycler pipeline to perform hybrid assembly based on QitanTech nanopore long-read data and Illumina short-read data. At first, we utilized total QitanTech and Illumina data to perform hybrid assembly for the six strains, respectively. However, strains RGT40-1, KP18-2073, and KP18-2110-2 were assembled into many contigs, far from complete genomes. Unicycler makes use of low quantities of long-read data to find the best paths through the assembly graph generated from short-read data assembly to produce a completed assembly (Wick et al., 2017). To eliminate the side effect of short-read sequences in QitanTech sequencing data in hybrid assembly, we filtered out the reads shorter than 5 kb from QitanTech sequences of the three strains to re-assemble with hybrid strategy. Finally, the results showed that the six bacterial genomes were nearly assembled successfully (Figure 4 and Supplementary Figure 1). In addition, few misassemblies and mismatches were detected in assembled genomes with hybrid strategy using Unicycler (Supplementary Figure 2). Further analysis found that the chromosomes of five strains were successfully resolved in right forms except ZXPA-20, which was consistent with the results of Flye assembly. In addition, some plasmids of the six strains were assembled incorrectly (Supplementary Table 4). For those plasmids that failed to assemble, such as a *tmexCD1-toprJ1*–bearing plasmid in RGT40-1, most of them also could not be directly resolved by hybrid assembly combine of ONT nanopore sequencing data and Illumina sequencing data. Single-molecule analysis is usually the last resort to deal with such plasmids (Li et al., 2018). Subsequently, we analyzed the result of hybrid assembly of strain ZXPA-20. The genome of ZXPA-20 was assembled into seven linear contigs with a total base of 7,084,385 bp, smaller than the reference genome size of 7,114,563 bp. The chromosome and the megaplasmid of ZXPA-20 shared many common IS*3-*like elements, which might be the cause of assembly failure. The megaplasmid of ZXPA-20 was assembled into three contigs, and the total length of the three contigs was smaller than reference megaplasmid pZXPA-20-602k (CP061724). Comparison analysis found that one IS*3*-like element with size 1,232 bp was lacked in the three contigs (Supplementary Figure 3). This further illustrated that IS elements were importantly negative factors in assembling bacterial genomes. In general, although, sometimes, the complete genome containing plasmids with complex structures could not be obtained directly by hybrid assembly combining of QitanTech nanopore sequencing data and Illumina sequencing data, in most cases, the complete bacterial genomes could be acquired with the help of QitanTech sequencing data.

### The Accuracy of Assembled Genomes Using QitanTech Nanopore Sequencing Data

The mean read accuracy of QitanTech nanopore sequencing raw reads is higher than 80%. The accuracy of assembled genomes was influenced by the sequencing coverage of total sequencing data sizes. Hence, we used different coverages of raw data to assemble the genomes and assessed the accuracy of assembled genome based on long-read data. We used the chromosome of strain XM9F202-2 as the test genome. The standard of successful assembly is that the chromosome was assembled into one circular closed contig. Genome accuracy was determined by dnadiffer in the MuMmer toolkit using complete XM9F202-2 chromosome (CP060811) as reference (Cornelius et al., 2021). The total bases of QitanTech sequencing data for strain XM9F202-2 were about 704 Mbp, which was about 200 X sequencing depth for the genome XM9F202-2. We randomly sampled the sequences from the 704-Mbp sequencing data using Seqkit and then assembled the sampled sequences with Flye. The minimum sequence data for assembling the genome successfully was about 24,769 reads of 145 Mbp with genome coverage 40 X. Subsequently, we used the complete chromosome of XM9F202-2 as reference to evaluate the accuracy of assembled genomes with different genome coverage. The results showed that the accuracy of assembled chromosome with 200 X sequencing depth had little improvement compared with the assembled chromosome with 40 X sequencing depth (99.27–99.31%) (Supplementary Table 5). The total SNPs between assembled chromosomes and complete reference chromosome decreased from 4,799 to 4,162 with the sequencing depth increasing (Supplementary Table 5). Hence, increase of the sequencing depth will not always result in improvement of the accuracy of assembled genome for QitanTech nanopore sequencing.

### The Genome Accuracy With Different Polishing Strategies

It seems impossible to obtain accurate bacterial genomes using QitanTech nanopore sequencing alone. Hence, we used highly accurate Illumina reads to polish the QitanTech long-read assemblies using different polishing strategies. Pilon, which has been widely used to polish the draft assembled genomes since it was initially released (Walker et al., 2014). It first utilized Illumina short-read data to map to the draft assembly; then, it improved the local accuracy of the sequence by correcting sequence errors, fixing misassemblies, and filling gaps. Meanwhile, applying Pilon iteratively had some benefits for improving the assembled genome (Walker et al., 2014). In the first round of polishing using Pilon, the accuracy of 200 X assembled chromosome of XM9F202-2 increased from 99.31 to 99.96%. Finally, the accuracy of chromosome XM9F202-2 assembled by QitanTech long-read data stopped increasing and reached 99.99% after four rounds of polishing (Supplementary Table 5). The total SNPs between polished chromosome after four rounds of polishing and the reference complete chromosome have only 139 (Supplementary Table 5). Although there is a significant improvement for genome accuracy after polishing using Pilon, the sporadic SNPs might affect the accuracy of downstream genomic analysis, such as core genetic phylogenetic analysis.

Recently, a fast and efficient genome polishing tool, NextPolish, was developed for polishing genome assemblies (Hu et al., 2020). One round of NextPolish polishing can significantly improve the bacterial genome accuracy compared with Pilon (Chen et al., 2021). Here, we still use the chromosome of strain XM9F202-2 assembled by QitanTech nanopore sequencing data as test sequence. After the first round of NextPolish polishing, the accuracy and SNPs of NextPolish-polished chromosome was 99.98% and 178, respectively (Supplementary Table 5). By contrast, the accuracy and SNPs of two rounds of NextPolish-polished chromosome were 99.98% and 210, respectively (Supplementary Table 5). Hence, one round of NextPolish polishing is sufficient to improve the quality of draft genome. Like Pilon, the NextPolish could not absolutely correct the long-read assembled genome using Illumina accurate reads. However, NextPolish was more efficient and easy at correcting sequence errors in QitanTech long-read assemblies compared to Pilon. All in all, both NextPolish and Pilon can significantly improve the quality of QitanTech long-read assemblies. In addition, the polished genome is enough to perform accurate genomic analyses of bacterial pathogens, such as identifying resistance genes and virulence genes. Meanwhile, we analyzed the accuracy of chromosome XM9F202-2 with hybrid assembly and found that it was more accurate than polished chromosome (Supplementary Table 5). Therefore, we suggested using a hybrid assembly strategy combining QitanTech sequences and short-read data to generate complete accurate bacterial genomes.

### Identification of Resistance Genes

**FIGURE 5:**
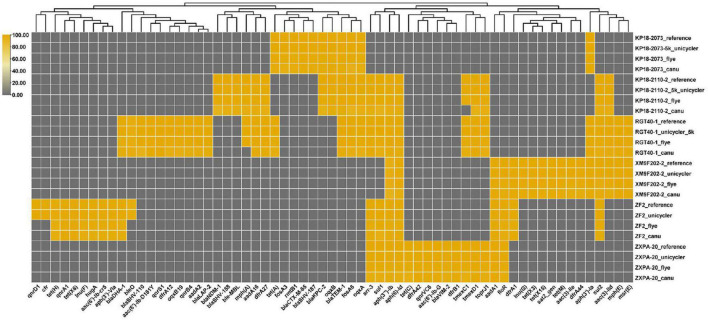
The distribution of antibiotic resistance genes. The resistance genes were identified using ResFinder 4.1.

We have obtained the draft or complete genomes of the six bacteria using different strategies (Flye, Canu, and Unicycler assembly). Then, we compared the effects of different genome assembly methods on the identification of resistance genes carried by these strains. The result showed that bacterial genomes assembled by Flye or Unicycler contained resistance genes consisting with the reference genomes except strain ZF2 (Figure 5). Genome analysis of strain ZF2 found that a 59 kb *cfr*-bearing plasmid pZF2-cfr was absent in genomes assembled by Flye and Unicycler using QitanTech sequencing data and Illumina sequencing data compared with complete genome of ZF2 (Peng et al., 2020). Subsequently, we used PCR to detect gene *cfr* in genome DNA of ZF2, but a negative result was received. We also tried to find the plasmid sequences of pZF2-cfr in the raw data of QitanTech sequencing data but found nothing. Hereby, we concluded that the plasmid pZF2-cfr was lost during enrichment culture before genomic DNA extraction. Meanwhile, this phenomenon demonstrated that QitanTech sequence has high resolution in identifying bacterial genome characteristics.

### Identification of a Fusion Plasmid in XM9F202-2

**FIGURE 6:**
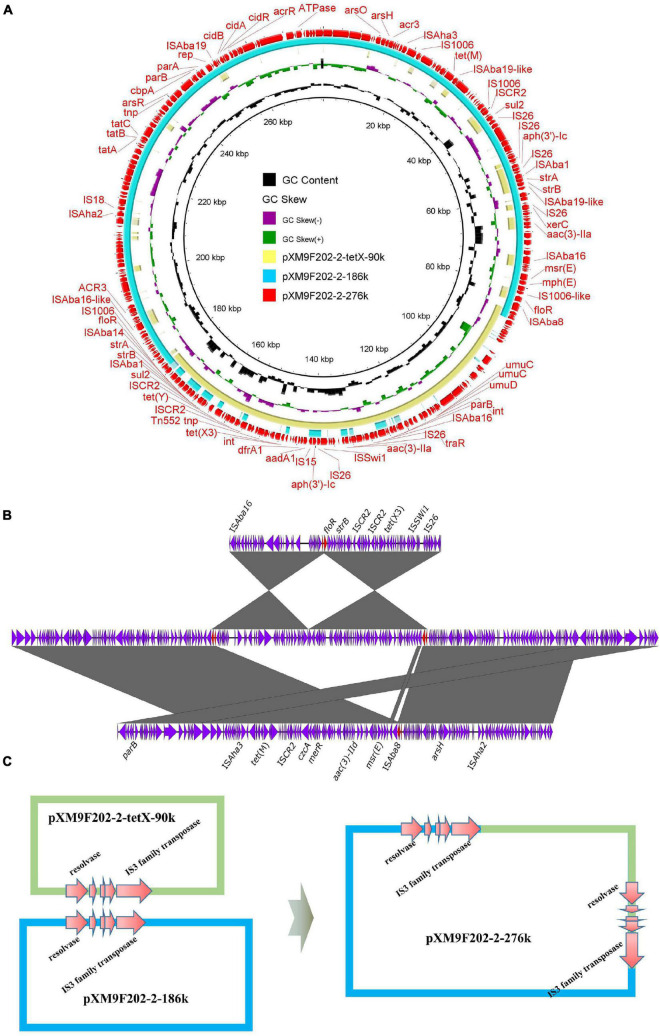
Mechanisms of plasmid fusions. **(A)** Comparing the fusion plasmid with its parental plasmids. **(B)** Line comparison of the fusion plasmid and its parental plasmids. **(C)** Schematic diagrams depicting the generation process of the fusion plasmid mediated by homologous regions.

In our previous study, we investigated the genome features of strain XM9F202-2 intensively, which harbored one chromosome and five plasmids (Li et al., 2021b). However, we found only four plasmids in assembled genomes of XM9F202-2 by Flye and Unicycler with QianTech sequencing data. Remarkably, one 276-kb plasmid, named pXM9F202-2-267k, of the four plasmids was almost the same as the total length of plasmids pXM9F202-2-tetX-90k and pXM9F202-2-186k in strain XM9F202-2. We speculated that a fusion event occurred between the two plasmids in the process of bacterial multiplication. Subsequently, we compared the structure of the 276-kb plasmid with plasmids pXM9F202-2-tetX-90k and pXM9F202-2-186k and found that the two small plasmids exactly covered the backbone of the large plasmid (Figure 6A). To prove the formation mechanism of the large fusion plasmid, we further explored the structure features of plasmid pXM9F202-2-267k in detail. Linear comparison between pXM9F202-2-267k and the two small plasmids pXM9F202-2-tetX-90k and pXM9F202-2-186k showed that they shared a homologous region (Figure 6B). Therefore, the plasmid fusion mechanism was highly possible resulting from homologous recombination (Figure 6C). Fusion plasmids were usually derived during the plasmids conjugation (Xie et al., 2018; Li et al., 2020a,b; Liu et al., 2020), which was a dynamic process with host changes. Here, we observed a fusion plasmid in different clones of the same strain and demonstrated that plasmid evolution happens all the time. Meanwhile, this phenomenon of plasmid fusion may promote the evolution of plasmids and increase the risk of transmission of resistant genes and virulence genes.

## Conclusion

In summary, this study comprehensively evaluated the ability of the novel QitanTech nanopore sequencing technology, the first long-read sequencers released in China, in bacterial genomes assembling with different assembling strategies. We found that most MDR bacterial genomes investigated here could be well resolved using QitanTech nanopore sequencing data. Meanwhile, complete accuracy bacterial genomes could be generated using hybrid assembly strategy combining of QitanTech nanopore sequencing data and high accurate Illumina data. Furthermore, many sequencing data processing tools for ONT nanopore sequences or PacBio SMRT sequences could directly deal with QitanTech nanopore sequencing data. As with ONT or PacBio sequencing, QitanTech sequencing data has high resolution in identifying complicated bacterial genomic features. With the advancement and continuous upgrades of QitanTech nanopore sequencing technology, its applications in diverse research and clinical settings deserve comprehensive investigations.

## Data Availability Statement

The genome sequences of QitanTech raw data were deposited in figshare database (https://figshare.com/articles/dataset/QitaTech_raw_data/15147570) for reference.

## Author Contributions

RL and ZW: conceptualization, writing—review and editing, and supervision. KP, RL, and YaL: methodology. KP, YY, and YuL: investigation. KP, SQ, and XY: data curation and visualization. KP and YY: writing—original draft preparation. All authors read and agreed to the published version of the manuscript.

## Conflict of Interest

The authors declare that the research was conducted in the absence of any commercial or financial relationships that could be construed as a potential conflict of interest.

## Publisher’s Note

All claims expressed in this article are solely those of the authors and do not necessarily represent those of their affiliated organizations, or those of the publisher, the editors and the reviewers. Any product that may be evaluated in this article, or claim that may be made by its manufacturer, is not guaranteed or endorsed by the publisher.

## Footnotes

1
http://rast.nmpdr.org/

2
https://cge.cbs.dtu.dk/services/
