# Supplementary material for: QitanTech Nanopore Long-Read Sequencing Enables Rapid Resolution of Complete Genomes of Multi-Drug Resistant Pathogens
Source: Front Microbiol. 2022 Mar 23;13:778659. doi: 10.3389/fmicb.2022.778659 (PMC8985760; doi:10.3389/fmicb.2022.778659)
Supplement: Supplementary file 1 [file Data_Sheet_1.docx]

**Supplementary Data**

Table S1. The basic information of strains in this study.

| Strains | KP18-2073 | KP18-2110-2 | RGT40-1 | XM9F202-2 | ZF2 | ZXPA-20 |
| --- | --- | --- | --- | --- | --- | --- |
| Species | *Klebsiella pneumoniae* | *Klebsiella pneumoniae* | *Klebsiella pneumoniae* | *Acinetobacter variabilis* | *Proteus cibarius* | *Pseudomonas putida* |
| Genome size | 6,013,359 | 5,838,658 | 5,889,759 | 3,481,134 | 4,306,177 | 7,114,630 |
| Chromosome size | 5,629,305 | 5,330,391 | 5,319,094 | 3,171,535 | 4,237,246 | 6,511,858 |
| Chromosome GC Content | 57.19% | 57.52 | 57.35% | 42.51% | 38.42% | 61.72% |
| Number of reads | 5 | 4 | 4 | 6 | 4 | 2 |
| GenBank accession No. | CP082023-CP082027 | CP084986-CP084989 | CP075548-CP075551 | CP060811-CP060816 | CP045008-CP045011 | CP061723-CP061724 |

Table S2. The sequence characteristics of QitanTech nanopore sequencing data.

|  | Total | KP18-2073 | KP18-2110-2 | RGT40-1 | XM9F202-2 | ZF2 | ZPXA-20 |
| --- | --- | --- | --- | --- | --- | --- | --- |
| Mean read length (bp) | 6041.3 | 6490.4 | 5376 | 5444.2 | 6108.1 | 6831.1 | 6088.4 |
| Mean read quality | 8.2 | 8.1 | 8 | 8.1 | 8.6 | 8.3 | 8.2 |
| Median read length (bp) | 5804 | 6342 | 4553 | 4858 | 6056 | 6859 | 5761 |
| Median read quality | 8.3 | 8.2 | 8.1 | 8.2 | 8.7 | 8.4 | 8.3 |
| Number of reads | 727854 | 146089 | 120343 | 98706 | 115291 | 72727 | 174698 |
| Read length N50 (bp) | 8314 | 8501 | 7920 | 8155 | 8150 | 8883 | 8277 |
| Longest read (bp) | 57037 | 47407 | 56215 | 52575 | 57037 | 51647 | 54480 |
| Total bases (bp) | 4397158294 | 948181467 | 646960864 | 537377872 | 704211220 | 496801810 | 1063625061 |
| Number, percentage and megabases of reads above quality cutoffs |  |  |  |  |  |  |  |
| Q5 | 727854 (100.0%) 4397.2Mb | 146089 (100.0%) 948.2Mb | 120343 (100.0%) 647.0Mb | 98706 (100.0%) 537.4Mb | 115291 (100.0%) 704.2Mb | 72727 (100.0%) 496.8Mb | 174698 (100.0%) 1063.6Mb |
| Q7 | 727732 (100.0%) 4397.2Mb | 146079 (100.0%) 948.2Mb | 120338 (100.0%) 647.0Mb | 98669 (100.0%) 537.4Mb | 115278 (100.0%) 704.2Mb | 72671 (99.9%) 496.8Mb | 174697 (100.0%) 1063.6Mb |
| Q10 | 87 (0.0%) 0.0Mb | 1 (0.0%) 0.0Mb | 0 (0.0%) 0.0Mb | 26 (0.0%) 0.0Mb | 14 (0.0%) 0.0Mb | 43 (0.1%) 0.0Mb | 3 (0.0%) 0.0Mb |
| Q12 | 20 (0.0%) 0.0Mb | 0 (0.0%) 0.0Mb | 0 (0.0%) 0.0Mb | 8 (0.0%) 0.0Mb | 4 (0.0%) 0.0Mb | 6 (0.0%) 0.0Mb | 2 (0.0%) 0.0Mb |
| Q15 | 2 (0.0%) 0.0Mb | 0 (0.0%) 0.0Mb | 0 (0.0%) 0.0Mb | 0 (0.0%) 0.0Mb | 0 (0.0%) 0.0Mb | 1 (0.0%) 0.0Mb | 1 (0.0%) 0.0Mb |

Table S3. The assembly statistics of six genomes using short-read data based on the SPAdes assembler.

| Strain_ID | Num_seqs | Sum_len (bp) | Min_len (bp) | Avg_len (bp) | Max_len (bp) |
| --- | --- | --- | --- | --- | --- |
| KP18-2073 | 332 | 5,901,962 | 78 | 17,777 | 469,818 |
| KP18-2110-2 | 160 | 5,774,587 | 78 | 36,091.2 | 395,718 |
| RGT40-1 | 170 | 5,780,090 | 78 | 34,000.5 | 412,411 |
| XM9F202-2 | 319 | 3,443,788 | 78 | 10,795.6 | 150,604 |
| ZF2 | 132 | 4,241,323 | 78 | 32,131.2 | 504,348 |
| ZXPA-20 | 181 | 7,014,184 | 78 | 38,752.4 | 564,155 |

Table S4. The assembly summary using Unicycler combining QitanTech sequencing data and Illumina sequencing data of the six strains.

| Strains | Contigs |
| --- | --- |
| KP18-2073 | contig1: 5629945bp circular chromosome  contig2: 237851bp circular plasmid  contig3: 123295bp linear plasmid  contig4: 54932bp circular plasmid false  contig5: 10060bp circular plasmid false  contig6: 5596bp circular plasmid  contig7: 2095bp circular plasmid |
| KP18-2110-2 | contig1: 5330281bp circular chromosome  contig2: 362034bp circular plasmid  contig3: 142244bp circular plasmid  contig4: 3883bp linear plasmid |
| ZXPA-20 | contig1: 6481731bp linear chromosome  contig2: 574072bp linear plasmid  contig3: 23659bp linear chromosome and plasmid  contig4: 3809bp linear unknow  contig5: 808bp linear unknow |
| ZF2 | contig1: 4237277bp circular chromosome  contig2: 52086bp linear plasmid  contig3: 3250bp linear unknow  contig4: 7081bp circular plasmid  contig5: 2683bp circular plasmid |
| XM9F202-2 | contig1: 3172342bp circular chromosome  contig2: 276672bp circular plasmid  contig3: 17496bp circular plasmid  contig4: 13130bp circular plasmid  contig5: 8840bp circular plasmid  contig6: 8593bp circular plasmid  contig7: 2301bp circular plasmid |
| RGT40-1 | contig1: 5319093bp circular chromosome  contig2: 248315bp linear palsmid  contig3: 181365bp circular plasmid  contig4: 78533bp circular plasmid  contig5: 30991bp circular plasmid false  contig6: 3883bp linear plasmid |

Table S5. The chromosome sequence accuracy of strain XM9F202-2 using different assembly methods with QitanTech sequencing data.

| [Sequences] |  | XM9F202-2_complete | unicycler | XM9F202-2_complete | Flye_40X | XM9F202-2_complete | Flye_200X | XM9F202-2_complete | polished_1R | XM9F202-2_complete | polished_4R | XM9F202-2_complete | NextPlion_1R | XM9F202-2_complete | NextPlion_2R |
| --- | --- | --- | --- | --- | --- | --- | --- | --- | --- | --- | --- | --- | --- | --- | --- |
|  | TotalSeqs | 1 | 1 | 1 | 1 | 1 | 1 | 1 | 1 | 1 | 1 | 1 | 2 | 1 | 1 |
|  | AlignedSeqs | 1(100.00%) | 1(100.00%) | 1(100.00%) | 1(100.00%) | 1(100.00%) | 1(100.00%) | 1(100.00%) | 1(100.00%) | 1(100.00%) | 1(100.00%) | 1(100.00%) | 1(50.00%) | 1(100.00%) | 1(100.00%) |
|  | UnalignedSeqs | 0(0.00%) | 0(0.00%) | 0(0.00%) | 0(0.00%) | 0(0.00%) | 0(0.00%) | 0(0.00%) | 0(0.00%) | 0(0.00%) | 0(0.00%) | 0(0.00%) | 1(50.00%) | 0(0.00%) | 0(0.00%) |
| [Bases] | TotalBases | 3171535 | 3172342 | 3171535 | 3153776 | 3171535 | 3154396 | 3171535 | 3170784 | 3171535 | 3170606 | 3171535 | 3188145 | 3171535 | 3170520 |
|  | AlignedBases | 3171535(100.00%) | 3171554(99.98%) | 3171476(100.00%) | 3153776(100.00%) | 3171533(100.00%) | 3154396(100.00%) | 3171533(100.00%) | 3170784(100.00%) | 3171533(100.00%) | 3170606(100.00%) | 3171533(100.00%) | 3170516(99.45%) | 3171533(100.00%) | 3170520(100.00%) |
|  | UnalignedBases | 0(0.00%) | 788(0.02%) | 59(0.00%) | 0(0.00%) | 2(0.00%) | 0(0.00%) | 2(0.00%) | 0(0.00%) | 2(0.00%) | 0(0.00%) | 2(0.00%) | 17629(0.55%) | 2(0.00%) | 0(0.00%) |
| [Alignments] | 1-to-1 | 3 | 3 | 3 | 3 | 3 | 3 | 4 | 4 | 4 | 4 | 4 | 4 | 4 | 4 |
|  | TotalLength | 3191651 | 3191680 | 3170746 | 3153783 | 3170801 | 3154403 | 3190891 | 3190908 | 3190891 | 3190730 | 3190891 | 3190640 | 3190891 | 3190644 |
|  | AvgLength | 1063883.67 | 1063893.33 | 1056915.33 | 1051261 | 1056933.67 | 1051467.67 | 797722.75 | 797727 | 797722.75 | 797682.5 | 797722.75 | 797660 | 797722.75 | 797661 |
|  | AvgIdentity | 99.99 | 99.99 | 99.27 | 99.27 | 99.31 | 99.31 | 99.96 | 99.96 | 99.99 | 99.99 | 99.98 | 99.98 | 99.98 | 99.98 |
|  | M-to-M | 3 | 3 | 5 | 5 | 4 | 4 | 5 | 5 | 5 | 5 | 5 | 5 | 5 | 5 |
|  | TotalLength | 3191651 | 3191680 | 3172208 | 3155241 | 3171534 | 3155136 | 3191624 | 3191641 | 3191624 | 3191463 | 3191624 | 3191373 | 3191624 | 3191377 |
|  | AvgLength | 1063883.67 | 1063893.33 | 634441.6 | 631048.2 | 792883.5 | 788784 | 638324.8 | 638328.2 | 638324.8 | 638292.6 | 638324.8 | 638274.6 | 638324.8 | 638275.4 |
|  | AvgIdentity | 99.99 | 99.99 | 99.27 | 99.27 | 99.31 | 99.31 | 99.96 | 99.96 | 99.99 | 99.99 | 99.98 | 99.98 | 99.98 | 99.98 |
|  | Breakpoints | 4 | 4 | 8 | 8 | 6 | 6 | 8 | 8 | 8 | 8 | 8 | 8 | 8 | 8 |
|  | Relocations | 0 | 0 | 1 | 2 | 1 | 2 | 1 | 2 | 1 | 2 | 1 | 2 | 1 | 2 |
|  | Translocations | 0 | 0 | 0 | 0 | 0 | 0 | 0 | 0 | 0 | 0 | 0 | 0 | 0 | 0 |
|  | Inversions | 0 | 0 | 0 | 0 | 0 | 0 | 0 | 0 | 0 | 0 | 0 | 0 | 0 | 0 |
|  | Insertions | 1 | 1 | 4 | 0 | 2 | 0 | 3 | 0 | 3 | 0 | 3 | 0 | 3 | 0 |
|  | InsertionSum | 18 | 796 | 1521 | 0 | 735 | 0 | 762 | 0 | 762 | 0 | 762 | 0 | 762 | 0 |
|  | InsertionAvg | 18 | 796 | 380.25 | 0 | 367.5 | 0 | 254 | 0 | 254 | 0 | 254 | 0 | 254 | 0 |
|  | TandemIns | 1 | 0 | 0 | 0 | 0 | 0 | 1 | 0 | 1 | 0 | 1 | 0 | 1 | 0 |
|  | TandemInsSum | 18 | 0 | 0 | 0 | 0 | 0 | 27 | 0 | 27 | 0 | 27 | 0 | 27 | 0 |
|  | TandemInsAvg | 18 | 0 | 0 | 0 | 0 | 0 | 27 | 0 | 27 | 0 | 27 | 0 | 27 | 0 |
| [SNPs] | TotalSNPs | 16 | 16 | 4799 | 4799 | 4162 | 4162 | 634 | 634 | 139 | 139 | 178 | 178 | 210 | 210 |
|  | TC | 1(6.25%) | 0(0.00%) | 169(3.52%) | 579(12.07%) | 116(2.79%) | 642(15.43%) | 39(6.15%) | 132(20.82%) | 21(15.11%) | 13(9.35%) | 7(3.93%) | 12(6.74%) | 13(6.19%) | 29(13.81%) |
|  | TG | 0(0.00%) | 0(0.00%) | 195(4.06%) | 1164(24.26%) | 126(3.03%) | 947(22.75%) | 14(2.21%) | 79(12.46%) | 5(3.60%) | 20(14.39%) | 16(8.99%) | 24(13.48%) | 31(14.76%) | 19(9.05%) |
|  | TA | 1(6.25%) | 2(12.50%) | 40(0.83%) | 43(0.90%) | 44(1.06%) | 24(0.58%) | 7(1.10%) | 22(3.47%) | 1(0.72%) | 4(2.88%) | 29(16.29%) | 11(6.18%) | 4(1.90%) | 5(2.38%) |
|  | GA | 2(12.50%) | 1(6.25%) | 106(2.21%) | 793(16.52%) | 642(15.43%) | 116(2.79%) | 53(8.36%) | 43(6.78%) | 11(7.91%) | 16(11.51%) | 15(8.43%) | 17(9.55%) | 8(3.81%) | 15(7.14%) |
|  | GC | 2(12.50%) | 0(0.00%) | 43(0.90%) | 40(0.83%) | 178(4.28%) | 195(4.69%) | 22(3.47%) | 7(1.10%) | 11(7.91%) | 7(5.04%) | 28(15.73%) | 11(6.18%) | 19(9.05%) | 31(14.76%) |
|  | GT | 1(6.25%) | 0(0.00%) | 174(3.63%) | 1048(21.84%) | 1073(25.78%) | 166(3.99%) | 42(6.62%) | 149(23.50%) | 20(14.39%) | 5(3.60%) | 12(6.74%) | 7(3.93%) | 33(15.71%) | 12(5.71%) |
|  | CT | 0(0.00%) | 2(12.50%) | 793(16.52%) | 106(2.21%) | 504(12.11%) | 147(3.53%) | 22(3.47%) | 32(5.05%) | 13(9.35%) | 21(15.11%) | 24(13.48%) | 16(8.99%) | 15(7.14%) | 8(3.81%) |
|  | CG | 0(0.00%) | 0(0.00%) | 1164(24.26%) | 195(4.06%) | 195(4.69%) | 178(4.28%) | 43(6.78%) | 53(8.36%) | 7(5.04%) | 11(7.91%) | 3(1.69%) | 5(2.81%) | 29(13.81%) | 13(6.19%) |
|  | CA | 6(37.50%) | 3(18.75%) | 252(5.25%) | 236(4.92%) | 947(22.75%) | 126(3.03%) | 79(12.46%) | 14(2.21%) | 21(15.11%) | 9(6.47%) | 11(6.18%) | 28(15.73%) | 21(10.00%) | 20(9.52%) |
|  | AG | 0(0.00%) | 1(6.25%) | 1048(21.84%) | 174(3.63%) | 166(3.99%) | 1073(25.78%) | 149(23.50%) | 42(6.62%) | 16(11.51%) | 11(7.91%) | 5(2.81%) | 3(1.69%) | 20(9.52%) | 21(10.00%) |
|  | AC | 0(0.00%) | 1(6.25%) | 579(12.07%) | 169(3.52%) | 24(0.58%) | 44(1.06%) | 132(20.82%) | 39(6.15%) | 9(6.47%) | 21(15.11%) | 17(9.55%) | 15(8.43%) | 12(5.71%) | 33(15.71%) |
|  | AT | 3(18.75%) | 6(37.50%) | 236(4.92%) | 252(5.25%) | 147(3.53%) | 504(12.11%) | 32(5.05%) | 22(3.47%) | 4(2.88%) | 1(0.72%) | 11(6.18%) | 29(16.29%) | 5(2.38%) | 4(1.90%) |
|  | TotalGSNPs | 8 | 8 | 1469 | 1469 | 1289 | 1289 | 524 | 524 | 40 | 40 | 51 | 51 | 60 | 60 |
|  | CA | 1(12.50%) | 0(0.00%) | 72(4.90%) | 80(5.45%) | 29(2.25%) | 208(16.14%) | 14(2.67%) | 27(5.15%) | 2(5.00%) | 0(0.00%) | 2(3.92%) | 1(1.96%) | 3(5.00%) | 1(1.67%) |
|  | CG | 1(12.50%) | 0(0.00%) | 342(23.28%) | 48(3.27%) | 24(1.86%) | 283(21.96%) | 60(11.45%) | 8(1.53%) | 2(5.00%) | 3(7.50%) | 8(15.69%) | 6(11.76%) | 5(8.33%) | 0(0.00%) |
|  | CT | 0(0.00%) | 0(0.00%) | 241(16.41%) | 40(2.72%) | 9(0.70%) | 7(0.54%) | 39(7.44%) | 40(7.63%) | 9(22.50%) | 8(20.00%) | 5(9.80%) | 0(0.00%) | 11(18.33%) | 8(13.33%) |
|  | GT | 1(12.50%) | 0(0.00%) | 4(0.27%) | 5(0.34%) | 68(5.28%) | 73(5.66%) | 8(1.53%) | 60(11.45%) | 2(5.00%) | 0(0.00%) | 0(0.00%) | 7(13.73%) | 11(18.33%) | 10(16.67%) |
|  | GA | 2(25.00%) | 0(0.00%) | 39(2.65%) | 253(17.22%) | 327(25.37%) | 26(2.02%) | 6(1.15%) | 20(3.82%) | 7(17.50%) | 5(12.50%) | 2(3.92%) | 1(1.96%) | 1(1.67%) | 3(5.00%) |
|  | GC | 0(0.00%) | 1(12.50%) | 48(3.27%) | 342(23.28%) | 208(16.14%) | 29(2.25%) | 24(4.58%) | 126(24.05%) | 3(7.50%) | 2(5.00%) | 6(11.76%) | 8(15.69%) | 8(13.33%) | 0(0.00%) |
|  | AT | 3(37.50%) | 0(0.00%) | 5(0.34%) | 4(0.27%) | 32(2.48%) | 203(15.75%) | 40(7.63%) | 39(7.44%) | 2(5.00%) | 0(0.00%) | 0(0.00%) | 5(9.80%) | 0(0.00%) | 5(8.33%) |
|  | AG | 0(0.00%) | 1(12.50%) | 39(2.65%) | 306(20.83%) | 26(2.02%) | 327(25.37%) | 20(3.82%) | 6(1.15%) | 5(12.50%) | 7(17.50%) | 1(1.96%) | 2(3.92%) | 10(16.67%) | 11(18.33%) |
|  | AC | 0(0.00%) | 1(12.50%) | 40(2.72%) | 241(16.41%) | 7(0.54%) | 9(0.70%) | 32(6.11%) | 128(24.43%) | 0(0.00%) | 2(5.00%) | 9(17.65%) | 10(19.61%) | 1(1.67%) | 2(3.33%) |
|  | TA | 0(0.00%) | 3(37.50%) | 306(20.83%) | 39(2.65%) | 203(15.75%) | 32(2.48%) | 27(5.15%) | 14(2.67%) | 0(0.00%) | 2(5.00%) | 1(1.96%) | 2(3.92%) | 8(13.33%) | 11(18.33%) |
|  | TC | 0(0.00%) | 0(0.00%) | 253(17.22%) | 39(2.65%) | 283(21.96%) | 24(1.86%) | 128(24.43%) | 32(6.11%) | 8(20.00%) | 9(22.50%) | 7(13.73%) | 0(0.00%) | 0(0.00%) | 8(13.33%) |
|  | TG | 0(0.00%) | 2(25.00%) | 80(5.45%) | 72(4.90%) | 73(5.66%) | 68(5.28%) | 126(24.05%) | 24(4.58%) | 0(0.00%) | 2(5.00%) | 10(19.61%) | 9(17.65%) | 2(3.33%) | 1(1.67%) |
|  | TotalIndels | 7 | 7 | 18485 | 18485 | 17636 | 17636 | 529 | 529 | 307 | 307 | 377 | 377 | 375 | 375 |
|  | T. | 0(0.00%) | 0(0.00%) | 4357(23.57%) | 224(1.21%) | 4230(23.99%) | 217(1.23%) | 66(12.48%) | 59(11.15%) | 61(19.87%) | 9(2.93%) | 114(30.24%) | 6(1.59%) | 69(18.40%) | 10(2.67%) |
|  | G. | 1(14.29%) | 0(0.00%) | 4361(23.59%) | 297(1.61%) | 4140(23.47%) | 96(0.54%) | 41(7.75%) | 65(12.29%) | 81(26.38%) | 13(4.23%) | 103(27.32%) | 9(2.39%) | 113(30.13%) | 6(1.60%) |
|  | C. | 4(57.14%) | 0(0.00%) | 4321(23.38%) | 111(0.60%) | 4482(25.41%) | 104(0.59%) | 85(16.07%) | 60(11.34%) | 83(27.04%) | 10(3.26%) | 71(18.83%) | 10(2.65%) | 102(27.20%) | 9(2.40%) |
|  | A. | 2(28.57%) | 0(0.00%) | 4685(25.34%) | 129(0.70%) | 4165(23.62%) | 202(1.15%) | 91(17.20%) | 62(11.72%) | 36(11.73%) | 14(4.56%) | 53(14.06%) | 11(2.92%) | 54(14.40%) | 12(3.20%) |
|  | .T | 0(0.00%) | 2(28.57%) | 129(0.70%) | 4685(25.34%) | 202(1.15%) | 4165(23.62%) | 62(11.72%) | 91(17.20%) | 9(2.93%) | 61(19.87%) | 9(2.39%) | 103(27.32%) | 6(1.60%) | 113(30.13%) |
|  | .A | 0(0.00%) | 4(57.14%) | 224(1.21%) | 4357(23.57%) | 104(0.59%) | 4482(25.41%) | 60(11.34%) | 85(16.07%) | 14(4.56%) | 36(11.73%) | 10(2.65%) | 71(18.83%) | 10(2.67%) | 69(18.40%) |
|  | .C | 0(0.00%) | 0(0.00%) | 297(1.61%) | 4361(23.59%) | 96(0.54%) | 4140(23.47%) | 65(12.29%) | 41(7.75%) | 10(3.26%) | 83(27.04%) | 11(2.92%) | 53(14.06%) | 9(2.40%) | 102(27.20%) |
|  | .G | 0(0.00%) | 1(14.29%) | 111(0.60%) | 4321(23.38%) | 217(1.23%) | 4230(23.99%) | 59(11.15%) | 66(12.48%) | 13(4.23%) | 81(26.38%) | 6(1.59%) | 114(30.24%) | 12(3.20%) | 54(14.40%) |
|  | TotalGIndels | 0 | 0 | 9693 | 9693 | 9368 | 9368 | 247 | 247 | 97 | 97 | 110 | 110 | 108 | 108 |
|  | C. | 0(0.00%) | 0(0.00%) | 2279(23.51%) | 61(0.63%) | 2339(24.97%) | 99(1.06%) | 32(12.96%) | 32(12.96%) | 26(26.80%) | 4(4.12%) | 39(35.45%) | 1(0.91%) | 39(36.11%) | 1(0.93%) |
|  | G. | 0(0.00%) | 0(0.00%) | 2355(24.30%) | 142(1.46%) | 2169(23.15%) | 40(0.43%) | 23(9.31%) | 40(16.19%) | 31(31.96%) | 3(3.09%) | 10(9.09%) | 0(0.00%) | 27(25.00%) | 1(0.93%) |
|  | A. | 0(0.00%) | 0(0.00%) | 2422(24.99%) | 98(1.01%) | 2372(25.32%) | 110(1.17%) | 10(4.05%) | 42(17.00%) | 7(7.22%) | 2(2.06%) | 28(25.45%) | 2(1.82%) | 28(25.93%) | 2(1.85%) |
|  | T. | 0(0.00%) | 0(0.00%) | 2290(23.63%) | 46(0.47%) | 2199(23.47%) | 40(0.43%) | 29(11.74%) | 39(15.79%) | 22(22.68%) | 2(2.06%) | 29(26.36%) | 1(0.91%) | 10(9.26%) | 0(0.00%) |
|  | .G | 0(0.00%) | 0(0.00%) | 61(0.63%) | 2279(23.51%) | 110(1.17%) | 2372(25.32%) | 32(12.96%) | 32(12.96%) | 3(3.09%) | 31(31.96%) | 1(0.91%) | 39(35.45%) | 0(0.00%) | 10(9.26%) |
|  | .C | 0(0.00%) | 0(0.00%) | 46(0.47%) | 2290(23.63%) | 40(0.43%) | 2199(23.47%) | 42(17.00%) | 10(4.05%) | 4(4.12%) | 26(26.80%) | 2(1.82%) | 28(25.45%) | 1(0.93%) | 27(25.00%) |
|  | .A | 0(0.00%) | 0(0.00%) | 142(1.46%) | 2355(24.30%) | 99(1.06%) | 2339(24.97%) | 40(16.19%) | 23(9.31%) | 2(2.06%) | 7(7.22%) | 0(0.00%) | 10(9.09%) | 2(1.85%) | 28(25.93%) |
|  | .T | 0(0.00%) | 0(0.00%) | 98(1.01%) | 2422(24.99%) | 40(0.43%) | 2169(23.15%) | 39(15.79%) | 29(11.74%) | 2(2.06%) | 22(22.68%) | 1(0.91%) | 29(26.36%) | 1(0.93%) | 39(36.11%) |


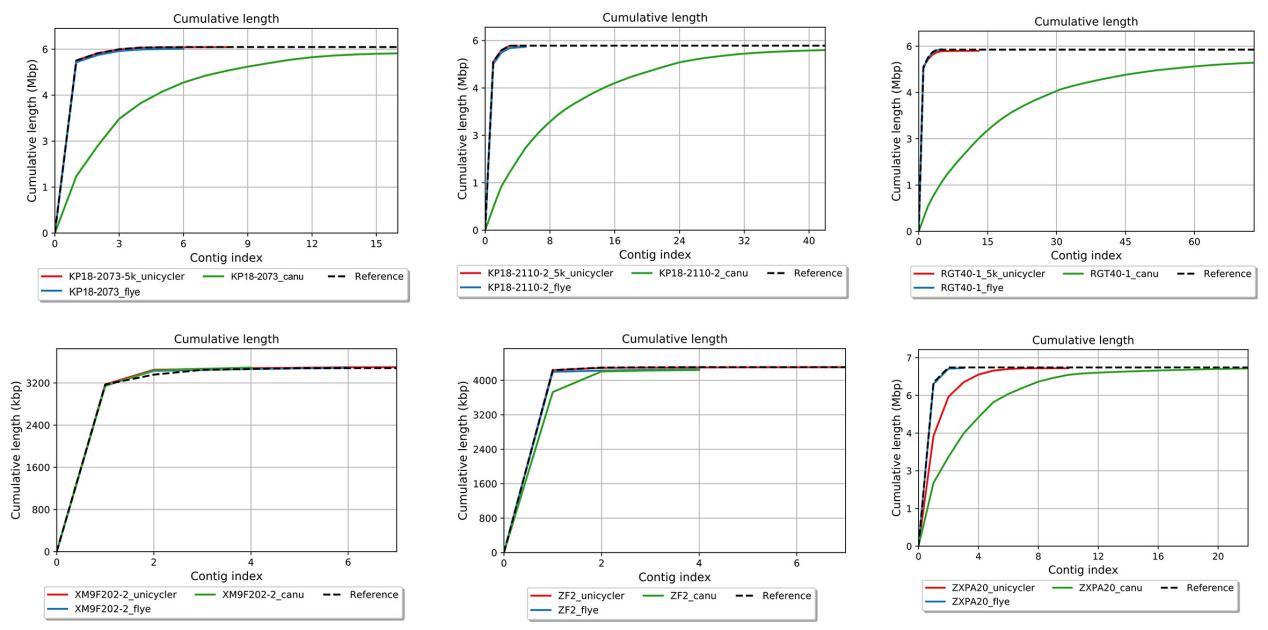


**Figure S1. Cumulative length of genomes for different assembly strategies.**

**
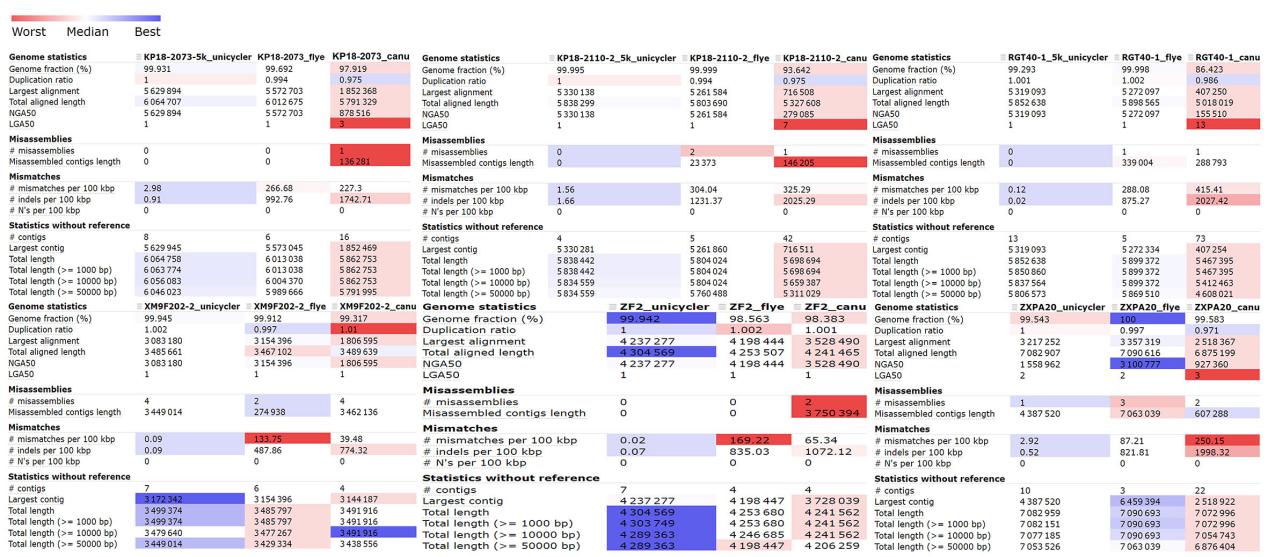
Figure S2. The quality assessment of assembled genomes using different methods.**

**
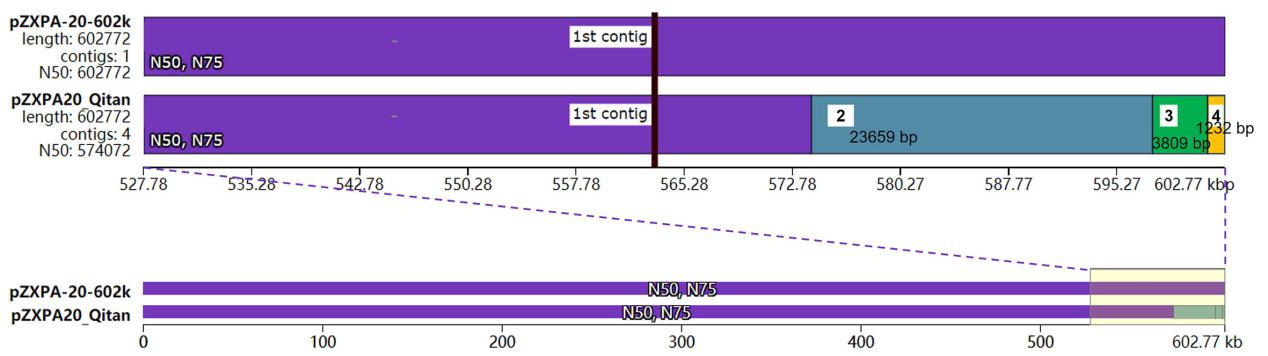
Figure S3. Comparative analysis of the megaplasmid pZXPA-20-602k with that assemblied with QitanTech sequencing data.**
